# Avianbase: a community resource for bird genomics

**DOI:** 10.1186/s13059-015-0588-2

**Published:** 2015-01-29

**Authors:** Lél Eöry, M Thomas P Gilbert, Cai Li, Bo Li, Alan Archibald, Bronwen L Aken, Guojie Zhang, Erich Jarvis, Paul Flicek, David W Burt

**Affiliations:** Department of Genomics and Genetics, The Roslin Institute and Royal (Dick) School of Veterinary Studies, University of Edinburgh, Easter Bush Campus, Midlothian, EH25 9RG UK; Centre for GeoGenetics, Natural History Museum of Denmark, University of Copenhagen, Øster Voldgade 5-7, 1350 Copenhagen, Denmark; Trace and Environmental DNA Laboratory, Department of Environment and Agriculture, Curtin University, Perth, Western Australia 6102 Australia; China National GeneBank, BGI-Shenzhen, Shenzhen, 518083 China; College of Medicine and Forensics, Xi’an Jiaotong University, Xi’an, 710061 China; European Molecular Biology Laboratory, European Bioinformatics Institute, Wellcome Trust Genome Campus, Hinxton, Cambridge CB10 1SD UK; Wellcome Trust Sanger Institute, Wellcome Trust Genome Campus, Hinxton, Cambridge CB10 1SA UK; Center for Social Evolution, Department of Biology, University of Copenhagen, Universitetsparken 15, Copenhagen, DK-2100 Denmark; Department of Neurobiology, Howard Hughes Medical Institute and Duke University Medical Center, Durham, NC 27710 USA

## Abstract

Giving access to sequence and annotation data for genome assemblies is important because, while facilitating research, it places both assembly and annotation quality under scrutiny, resulting in improvements to both. Therefore we announce Avianbase, a resource for bird genomics, which provides access to data released by the Avian Phylogenomics Consortium.

Access to complete genome sequences provides the first step towards the understanding of the biology of organisms. It is the template that underpins the phenotypic characteristics of individuals and ultimately separates species due to the accumulation and fixation of mutations over evolutionary timescales. In terms of the available genomic datasets for species, birds, as our more distant relatives, have been historically underrepresented. The high cost of sequencing and annotation in the past led to a bias towards accumulating data for species that are either established model organisms or economically significant (that is, chicken, turkey and duck, representing two sister orders within the Galloanseriformes clade from the large and diverse phylogeny of birds). The recent release of genome assemblies and initial predictions of protein-coding genes [1-4] for 44 bird species, including representatives from all major branches of the bird phylogeny, is, therefore, highly significant.

One of the major challenges with the release of this number of newly sequenced genomes and the many more to come [5] is how to make these available to the various research communities in a way that supports basic research. Providing access to the sequences and initial annotations in the format of text files will limit the potential usage of the data as they require significant resources, including bioinformatics personnel and computer infrastructure in place to access and mine - for example, searching for genes belonging to certain protein families or searching for orthologous genes. These overheads pose a serious bottleneck that can hinder research and requires concerted action by the relevant research communities.

Once genomes are submitted to public databases, genome-wide annotations are frequently generated and released either via the Ensembl project [6] or by the National Center for Biotechnology Information [7] and sequence and annotation are then made visually available online in integrated views via the Ensembl or the University of California Santa Cruz (UCSC) genome browsers [8]. These systems provide search facilities, sequence alignment tools like BLAT/BLAST and various analysis tools to facilitate subsetting and computational retrieval of the data, including UCSC’s Table Browser or Ensembl’s Perl and REST APIs and BioMart system.

While these systems have become almost indispensable for research, not all sequenced genomes are annotated and displayed in genome browsers. Full genome annotation remains time consuming and resource intensive: a full evidence-based Ensembl genebuild takes approximately 4 months. Thus, the list of species represented is currently limited and depends on various factors, including the completeness of the assembled genome sequence and the overall demand in the scientific community for the resources, including whether the species is a model organism (for example, human or mouse), economically important (for example, farmed animals) or of specific phylogenetic interest. Many of the recently sequenced bird genomes do not obviously fall within these categories.

## Bird genomics resource using Ensembl infrastructure

In order to support bird genomics by making the sequence and gene predictions generated by the Avian Phylogenomics Consortium (APC) more broadly available, as well as to support the research and conclusions in the published companion papers, we decided to make the initial data available within the Ensembl framework. We chose to use Ensembl for many reasons. First, Ensembl’s open-access data model and open-source software infrastructure make it possible to reuse their data and employ their source code for our purposes with minimal customizations. The software infrastructure includes various analysis pipelines and implements the genome browser interface with its unique tool-set. Second, the eHive analysis workflow management system [9] developed by the Ensembl team provides support for various computer infrastructures and greatly simplifies the tasks related to job management. Third, Ensembl runs a two tier user support system that quickly and efficiently resolves, beside many things, system-related problems via email to its helpdesk or through access to its developers through a dedicated mailing list. Finally, the modular design of the existing software infrastructure makes it possible to extend the analysis pipelines with new software or to create pipelines for new data types, to provide services matching the available data and/or computer infrastructure, and most importantly to scale-up data loading and analyses to a multispecies level.

**Figure 1:**
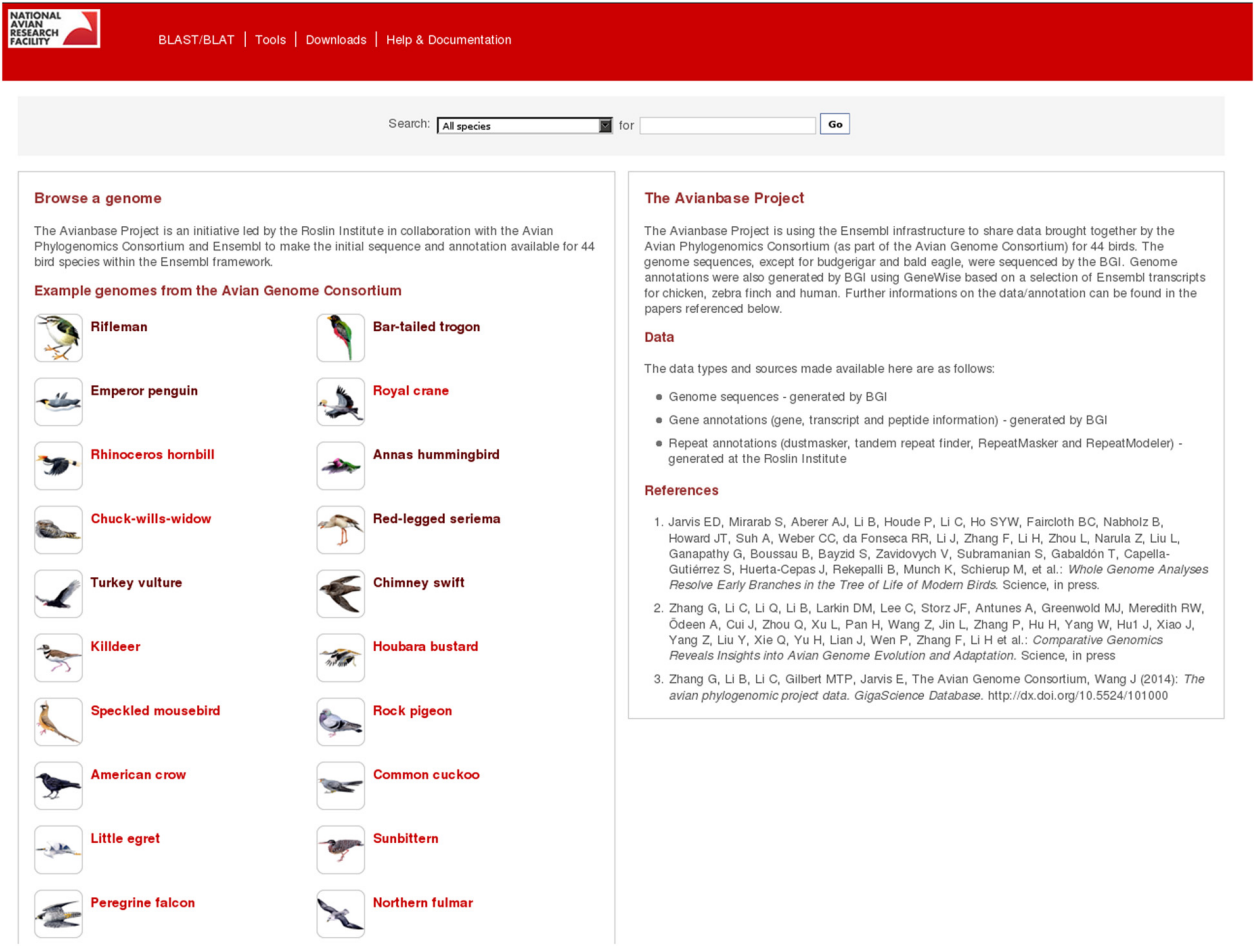
**Avianbase: genome portal for bird genomics using the Ensembl infrastructure.**

**Figure 2:**
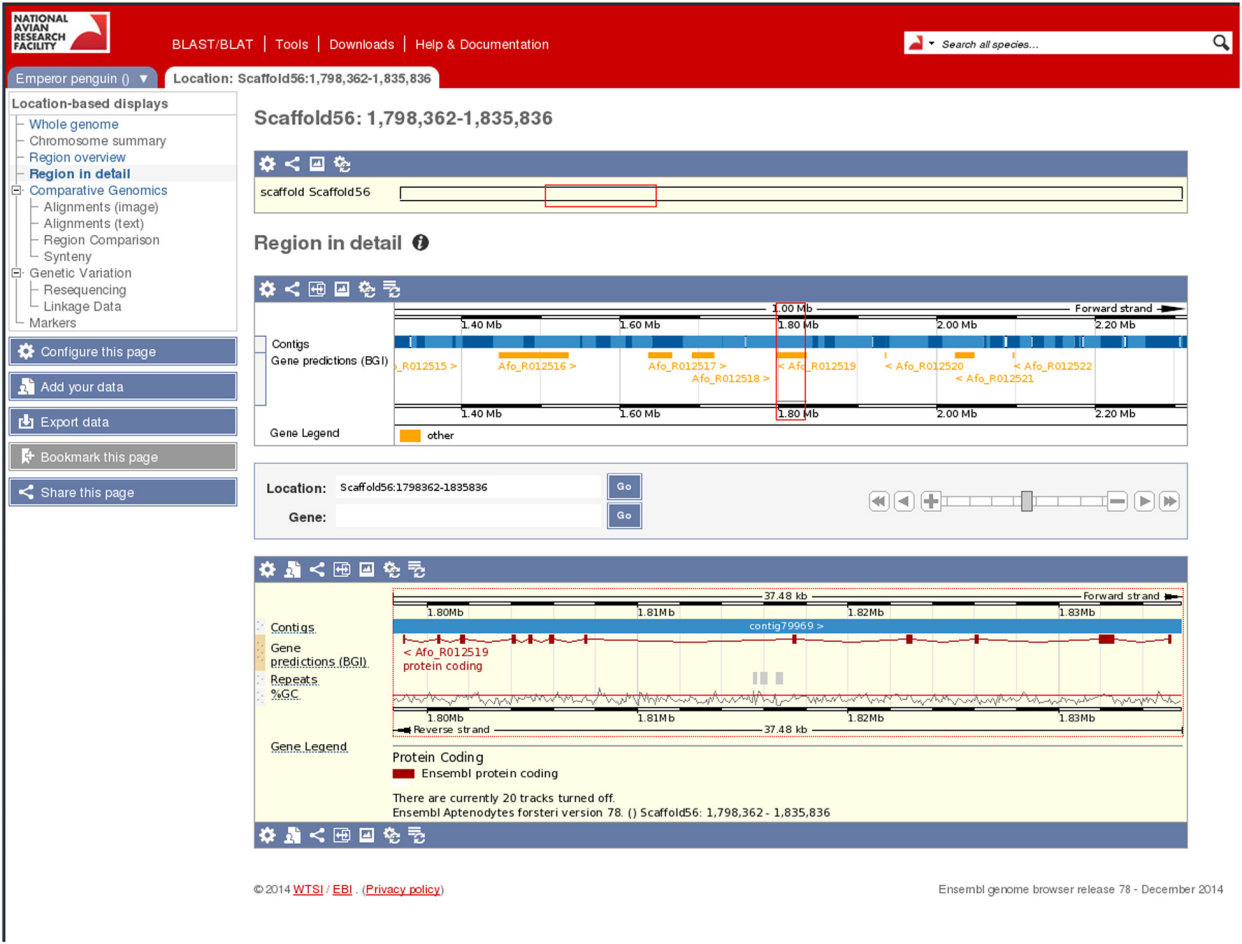
**Location view with example gene model and repeat annotation for Emperor penguin using the Ensembl Genome Viewer.**

Here we provide Avianbase, an Ensembl-based resource that is primarily built by and for the bird research communities to share and improve the existing data and annotation made available by the consortium. In its current form this Ensembl instance provides unique access to 44 newly sequenced bird genomes (Figure 1). The data include the genome assemblies generated by BGI, full repeat annotations using dustmasker [10], tandem repeat finder [11], homology-based repeat identification with RepeatMasker [12] and *de novo* repeat identification with RepeatModeler [13] as well as GeneWise [14] gene predictions created by the BGI and based on a set of selected transcripts from the chicken, zebra finch and human Ensembl genebuilds [1-4] (Figure 2). We also include within Avianbase a mirror of four relevant Ensembl core databases: chicken, turkey, duck and zebra finch, as some of these birds served as templates for the gene predictions and also because this set of 48 birds is the subject of the research described in many of the companion papers to the main APC papers [1,2]. In addition to providing visual displays of the sequences, gene models, transcripts and translations, we also provide indexed search facilities for these birds and BLAST access to the genomic data as well as links to the original data files [15]. Users can also upload and display their own data along with the default annotations. Future support for data mining and analysis is also planned by allowing access to the data via BioMart or via the Perl API and we are actively considering how to provide these options.

## Conclusions

Although at present the sequence data and annotations available on our site are limited to what was released by the APC, our bird portal can serve as a medium to support avian research in many ways.

One of our aims is to use this broad sample of available bird genomes to generate an improved functional map of selectively constrained sites for bird genomes in a genome-wide manner and in a functional category-independent way. This map will greatly improve our ability to link causative variants with genomic locations and so link certain genotypes with observed phenotypes. In the past, detailed maps of this kind were only available for mammals [16] and now we have the opportunity to greatly enhance avian research, especially for species for which variation data are already available (see, for example, [17]).

Our bird portal can be tailored to the needs of the individual bird research communities. It can list available resources and support collaboration within and between research teams by providing and sharing data that can be used to improve the assembly (resequencing projects) or the annotation (variation and transcriptome data) for the genome of interest. We encourage these communities to contact us (avianbase@ed.ac.uk) and suggest ways for improvements that can benefit their research.

Avianbase, our Ensembl-based bird resource, is available at http://avianbase.narf.ac.uk and is hosted within the National Avian Research Facility (NARF), UK [18], which aims to support the study of avian biology, genetics, infection and disease.

## Abbreviations

APC: Avian Phylogenomics Consortium
NARF: National Avian Research Facility
UCSC: University of California Santa Cruz
